# Evolution and Comparative Physiology of Luqin-Type Neuropeptide Signaling

**DOI:** 10.3389/fnins.2020.00130

**Published:** 2020-02-18

**Authors:** Luis Alfonso Yañez-Guerra, Maurice R. Elphick

**Affiliations:** School of Biological and Chemical Sciences, Faculty of Science and Engineering, Queen Mary University of London, London, United Kingdom

**Keywords:** luqin, cardio-excitatory peptide, RYamides, RWamides, neuropeptide evolution, G-protein coupled receptors

## Abstract

Luqin is a neuropeptide that was discovered and named on account of its expression in left upper quadrant cells of the abdominal ganglion in the mollusc *Aplysia californica*. Subsequently, luqin-type peptides were identified as cardio-excitatory neuropeptides in other molluscs and a cognate receptor was discovered in the pond snail *Lymnaea stagnalis.* Phylogenetic analyses have revealed that orthologs of molluscan luqin-type neuropeptides occur in other phyla; these include neuropeptides in ecdysozoans (arthropods, nematodes) that have a C-terminal RYamide motif (RYamides) and neuropeptides in ambulacrarians (echinoderms, hemichordates) that have a C-terminal RWamide motif (RWamides). Furthermore, precursors of luqin-type neuropeptides typically have a conserved C-terminal motif containing two cysteine residues, although the functional significance of this is unknown. Consistent with the orthology of the neuropeptides and their precursors, phylogenetic and pharmacological studies have revealed that orthologous G-protein coupled receptors (GPCRs) mediate effects of luqin-type neuropeptides in spiralians, ecdysozoans, and ambulacrarians. Luqin-type signaling originated in a common ancestor of the Bilateria as a paralog of tachykinin-type signaling but, unlike tachykinin-type signaling, luqin-type signaling was lost in chordates. This may largely explain why luqin-type signaling has received less attention than many other neuropeptide signaling systems. However, insights into the physiological actions of luqin-type neuropeptides (RYamides) in ecdysozoans have been reported recently, with roles in regulation of feeding and diuresis revealed in insects and roles in regulation of feeding, egg laying, locomotion, and lifespan revealed in the nematode *Caenorhabditis elegans.* Furthermore, characterization of a luqin-type neuropeptide in the starfish *Asterias rubens* (phylum Echinodermata) has provided the first insights into the physiological roles of luqin-type signaling in a deuterostome. In conclusion, although luqin was discovered in *Aplysia* over 30 years ago, there is still much to be learnt about luqin-type neuropeptide signaling. This will be facilitated in the post-genomic era by the emerging opportunities for experimental studies on a variety of invertebrate taxa.

## Introduction

Neuropeptides are evolutionarily ancient neuronal signaling molecules that typically exert their effects on target cells by binding to cognate G-protein coupled receptors (GPCRs) (Elphick et al., 2018; Jékely et al., 2018). Phylogenetic studies have revealed that the evolutionary origin of at least 30 neuropeptide signaling systems can be traced back to the bilaterian common ancestor of deuterostomes and protostomes. However, some neuropeptide signaling systems have been lost in one or more bilaterian phyla/sub-phyla (Jékely, 2013; Mirabeau and Joly, 2013; Elphick et al., 2018). Luqin-type neuropeptide signaling, which is the focus of this review, is one of the bilaterian neuropeptide signaling systems that have been lost in chordates/vertebrates. This may in part explain why less is known about luqin-type neuropeptide signaling than other bilaterian neuropeptide systems that have been retained in vertebrates. Nevertheless, several advances in our knowledge of the evolution and comparative physiology of luqin-type signaling in invertebrates have been made recently and therefore writing of this the first review article on luqin-type neuropeptide signaling is timely.

The neuropeptide luqin and its cognate GPCR were first discovered in molluscs (Shyamala et al., 1986; Fujimoto et al., 1990; Tensen et al., 1998). Subsequently, luqin-like neuropeptides known as RYamides, on account of a C-terminal Arg-Tyr-NH_2_ motif, were discovered in the arthropod *Cancer borealis* (Li et al., 2003). Furthermore, receptors for RYamides were identified in the fruitfly *Drosophila melanogaster* and in the red flour bettle *Tribolium castaneum* (Collin et al., 2011; Ida et al., 2011). In 2013, evidence that molluscan luqin-type signaling and arthropod RYamide-type signaling are orthologous was reported. Thus, use of pairwise-based clustering methods revealed that luqin precursors and RYamide precursors form part of the same protein cluster (Jékely, 2013). Furthermore, phylogenetic analysis of G-protein coupled neuropeptide receptors revealed that molluscan luqin receptors are orthologs of arthropod RYamide receptors (Mirabeau and Joly, 2013). In addition, these two studies also reported for the first time the discovery of a luqin-type signaling system in the nematode *Caenorhabditis elegans*, which has subsequently been functionally characterized experimentally (Jékely, 2013; Mirabeau and Joly, 2013; Ohno et al., 2017).

Importantly, phylogenetic analysis of transcriptome/genome sequence data has revealed that while luqin-type signaling has been lost in vertebrates and other chordates (urochordates, cephalochordates), genes encoding luqin-type precursors and receptors are present in ambulacrarian deuterostomes (echinoderms and hemichordates) (Jékely, 2013; Mirabeau and Joly, 2013). Thus, it was established for the first time that the evolutionary origin of luqin-type neuropeptide signaling predates the divergence of protostomes and deuterostomes, but with differential loss in the deuterostome branch of the Bilateria. Luqin-type neuropeptides in ambulacrarians are characterized by a predicted C-terminal RWamide motif (Elphick and Mirabeau, 2014; Rowe et al., 2014; Semmens et al., 2016). Furthermore, biochemical characterization of luqin-type neuropeptide signaling in the starfish *Asterias rubens* (phylum Echinodermata) demonstrated that a neuropeptide with the confirmed structure EKGRFPKFMRW-NH_2_ acts as a ligand for two luqin-type receptors in this species (Yañez-Guerra et al., 2018). Thus, the luqin-type neuropeptides in spiralian protostomes, the luqin-type RYamides in ecdysozoan protostomes, and the luqin-type RWamides in ambulacrarian deuterostomes have been unified as members of a bilaterian family of neuropeptides (Jékely, 2013; Mirabeau and Joly, 2013; Yañez-Guerra et al., 2018). It is against the backdrop of these important recent findings that we review here our knowledge of the evolution and comparative physiology of luqin-type neuropeptide signaling.

## The Discovery and Functional Characterization of Luqin-Type Neuropeptide Signaling in Molluscs

**FIGURE 1:**
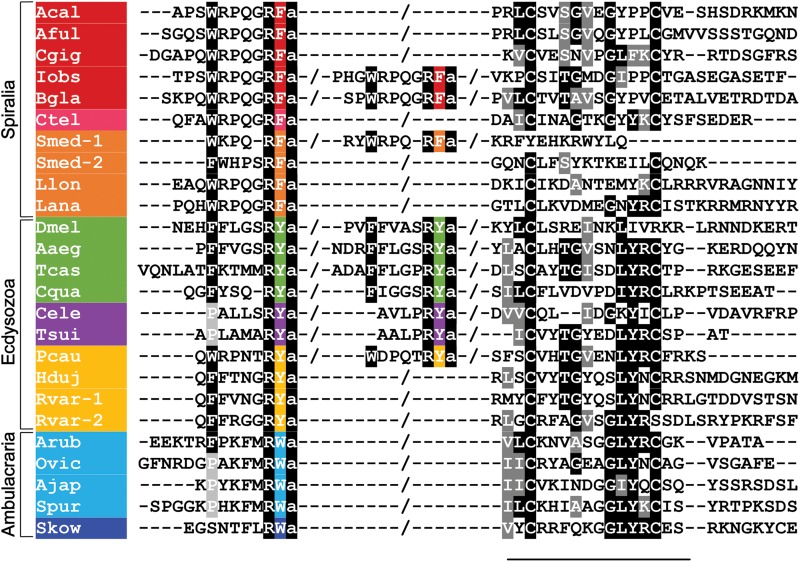
Alignment of the N-terminal neuropeptide-containing and C-terminal regions of luqin-type precursor proteins in bilaterians. Conserved residues are highlighted in black or gray. The C-terminal residues of the luqin-type neuropeptides and species names are highlighted in phylum-specific colors: red (Mollusca), pink (Annelida), orange (Platyhelminthes, Brachiopods, and Nemerteans), green (Arthropoda), purple (Nematoda), yellow (Priapulida and Tardigrada), light blue (Echinodermata), and dark blue (Hemichordata). Species names are as follows: Acal (*Aplysia californica*), Aful (*Achatina fulica*), Cgig (*Crassostrea gigas*), Iobs (*Ilyanasa obsoleta*), Bgla (*Biomphalaria glabrata*), Ctel (*Capitella teleta*), Smed (*Schmidtea mediterranea*), Lana (*Lingula anatina*), Llon (*Lineus longissimus)*, Dmel (*Drosophila melanogaster*), Aaeg (*Aedes aegypti*), Tcas (*Tribolium castaneum*), Cqua (*Cherax quadricarinatus*) Tsui (*Trichuris suis*), Cele (*Caenorhabditis elegans*), Pcau (Priapulus caudatus), Hduj (*Hypsibius dujardini*), Rvar (*Ramazzottius varieornatus*), Arub (*Asterias rubens*), Ovic (*Ophionotus victoriae*), Ajap (*Apostichopus japonicus*), Spur (*Strongylocentrotus purpuratus*), Skow (*Saccoglossus kowalevskii*). The sequences used for this alignment were reported in Koziol et al. (2016); Koziol (2018), Yañez-Guerra et al. (2018), and De Oliveira et al. (2019).

The discovery of the neuropeptide luqin was enabled by identification of transcripts expressed in the L5 neuron of the mollusc *Aplysia californica*. This was decades before the development of contemporary single cell transcriptomic methodologies and was facilitated by the large size of the cell body of the L5 neuron (Shyamala et al., 1986). Analysis of the expression of the cardio-excitatory neuropeptide FMRFamide in the central nervous system of *A. californica* using antibodies to FMRFamide revealed immunostaining in many neurons, including the L5 neuron that is located in the upper quadrant of the left abdominal ganglion (Brown et al., 1985; Schaefer et al., 1985). However, it was found that the FMRFamide gene is not expressed in the L5 neuron. Therefore, to determine the identity of the neuropeptide(s) responsible for FMRFamide-like immunoreactivity in L5, transcripts expressed in this neuron were sequenced. Sequencing of a transcript named L5-67 revealed that it encodes a 112 amino acid residue protein comprising an N-terminal signal peptide followed by a neuropeptide with a predicted C-terminal RFamide motif, which therefore could be cross-reactive with FMRFamide antibodies (Shyamala et al., 1986). Subsequent biochemical analysis revealed that processing of this precursor gives rise to the amidated decapeptide APSWRPQGRF-NH_2_, which was named luqin because it is expressed in the left upper quadrant cells of the abdominal ganglion (Aloyz and DesGroseillers, 1995). Furthermore, a 76 amino acid peptide corresponding to the region of the precursor protein C-terminal to the luqin neuropeptide was also detected and named proline-rich mature peptide (PRMP) (Aloyz and DesGroseillers, 1995). PRMP contains two cysteines separated by a 10 amino acid-residue sequence and subsequent studies have revealed that this is an evolutionarily conserved feature of luqin-type precursor proteins (Figure 1) (Jékely, 2013; Mirabeau and Joly, 2013; Yañez-Guerra et al., 2018).

In addition to luqin and PRMP, a mass spectrometric survey of the left upper quadrant neurons revealed that two other peptides are derived from the *A. californica* luqin precursor, which were named luqin-B and luqin-C (Li et al., 1998). The luqin-B fragment contains part of the mature luqin neuropeptide and the luqin-C fragment contains a shorter version of PRMP (Li et al., 1998), but it is not known if PRMP, luqin-B, or luqin-C are biologically active molecules. However, it has been shown that alternative splicing by exon skipping of one of the exons of the gene encoding the luqin precursor results in a frame shift and production of a precursor protein comprising the complete mature amidated decapeptide luqin and a short C-terminal region that does not contain the two cysteines characteristic of PRMP. This suggests that PRMP may not be essential for biosynthesis of the mature luqin neuropeptide. Still, from a functional perspective, it is noteworthy that while the full-length transcript is widely expressed in *A. californica*, the alternative transcript is specifically expressed in the kidney (Angers and DesGroseillers, 1998).

Sequencing of transcripts encoding luqin-type precursors and mass spectrometric identification of neuropeptides derived from them has revealed a high level of sequence conservation of luqin-type neuropeptides in molluscs. For example, in the giant snail *Achatina fullica*, a luqin-type peptide was identified as an amidated undecapeptide SGQSWRPQGRF-NH_2_, the C-terminal region of which (underlined) is identical to *Aplysia* luqin (Fujimoto et al., 1990), and in the pond snail *Lymnaea stagnalis* the luqin-type peptide TPHWRPQGRF-NH_2_ was identified. It is noteworthy that the luqin precursors in *A. californica*, *L. stagnalis*, and *A. fullica* comprise a single luqin-type neuropeptide, whereas in some other gastropod molluscs, such as the eastern mudsnail *Ilyanassa obsoleta* and the freshwater snail *Biomphalaria glabrata*, the precursor comprises two luqin-type neuropeptides (Figure 1). The first insight into the characteristics of luqin receptors was made with the discovery that the luqin-type peptide TPHWRPQGRF-NH_2_ is a ligand for an orphan G protein-coupled receptor, GRL106, in the pond snail *L. stagnalis.* It was also reported that this receptor is closely related to vertebrate tachykinin receptors and the *Drosophila* neuropeptide-Y-type receptor (Tensen et al., 1998).

Insights into the physiological roles of luqin-type neuropeptides were facilitated by the identification of a luqin-type neuropeptide in the snail *A. fullica*—a pulmonate gastropod. The peptide was discovered on account of its bioactivity as a cardioactive peptide that causes an increase in the frequency of beating when applied to auricle preparations from *A. fullica*. Hence, this peptide was named *Achatina* cardioexcitatory peptide (ACEP) (Fujimoto et al., 1990). However, ACEP also affects other muscle systems in *A. fullica*, increasing the amplitude of tetanic contraction of the penis retractor muscle and buccal muscle in response to electrical stimulation. Furthermore, ACEP was found to induce depolarization and rhythmic firing of a motor neuron (B4) that innervates buccal muscle (Fujimoto et al., 1990), an effect indicative of a physiological role in regulation of feeding behavior. Consistent with findings from *A. fullica*, the luqin-type peptide identified as a ligand for the *Lymnaea* receptor GRL106 was also found to be cardioexcitatory in this pulmonate gastropod species. Hence, the peptide was named *Lymnaea* cardioexcitatory peptide (LyCEP). Furthermore, the sequence similarity that LyCEP and ACEP share with *Aplysia* luqin was noted (Tensen et al., 1998). In accordance with the cardioexcitatory actions of LyCEP, immunohistochemical analysis revealed that LyCEP-immunoreactivity is present in nerve fibers ending in the pericardial cavity of the heart, indicating that the peptide is released into the pericardial cavity as a neurohormone (Tensen et al., 1998). However, LyCEP is not, as its name implies, specifically a cardioactive peptide because it is also expressed in nerve fibers associated with inhibition of the egg-laying hormone-producing caudodorsal cells in *L. stagnalis.* Accordingly, transcripts encoding the LyCEP receptor are present in the caudodorsal cells and LyCEP causes hyperpolarization of these cells *in vitro* (Tensen et al., 1998).

Analysis of the expression of the luqin precursor in the ophistobranch gastropod *A. californica* revealed transcripts in approximately 100 neurons of the central nervous system, but predominantly in neurons that innervate the circulatory and reproductive systems. In peripheral tissues, transcripts were detected in the intestine and in the kidneys (Giardino et al., 1996). Whole mount immunolabeling experiments with an antibody directed against the luqin precursor revealed immunoreactive fibers in different regions of the circulatory system of *Aplysia*, including the auricle, the ventricle, and the aorta. In the reproductive system, immunoreactive fibers were detected in the small and large hermaphroditic ducts and in the ovotestis (Giardino et al., 1996). The kidney also displayed immunoreactivity, located on the inner surface of the kidney wall. Strong immunoreactivity was also seen in neurites located in a large nerve associated with muscles of the renal pore, a sphincter that controls urine efflux (Angers et al., 2000). Altogether, these findings suggest roles of luqin-type peptides in regulation of the reproductive and circulatory systems, in fluid mobilization, and water homeostasis in gastropod molluscs.

While what is known about luqin expression and function in molluscs is largely based on experimental studies on selected gastropod species, as detailed above, genes/transcripts encoding luqin-type precursors have been identified in other gastropod species and in species belonging to other molluscan classes, including Bivalvia, Scaphopoda, Cephalopoda, Monoplacophora, Polyplacophora, Chaetodermomorpha, and Neomeniomorpha (De Oliveira et al., 2019). Thus, the occurrence of luqin-type neuropeptide precursors throughout the phylum Mollusca has been established, providing a basis for extending analysis of luqin-type neuropeptide function beyond gastropods to other classes.

## Luqin-Type Signaling in Annelids

Luqin-type precursors with a similar organization to those in molluscs have been identified in annelids. Analysis of genomic sequence data from *Capitella teleta* revealed the occurrence of a luqin-type precursor comprising the predicted mature peptide QFAWRPQGRF-NH_2_ (Veenstra, 2011) (Figure 1). Later, a partial precursor transcript was identified in the annelid *Platynereis dumerilii* and the structure of the luqin-type peptide derived from this precursor was confirmed as WRPQGRF-NH_2_ using mass spectrometry (Conzelmann et al., 2013). Furthermore, a transcript encoding a luqin-type receptor was identified in *P. dumerilii* and pharmacological characterization of this receptor demonstrated that the luqin-type peptide WRPQGRF-NH_2_ acts as a ligand for this receptor (Bauknecht and Jékely, 2015). Currently, there are no data available that provide insights into the physiological roles of luqin-type neuropeptides in annelids.

## Luqin-Type Signaling in Other Spiralians

Analysis of genome/transcriptome sequence data has revealed the occurrence of genes/transcripts encoding luqin-type precursors in a variety of species belonging to the phylum Platyhelminthes. For example, in the planarian *Schmidtea mediterranea*, an expanded family of genes encoding four luqin-type precursors has been identified (Koziol et al., 2016). The predicted neuropeptides derived from these precursors share sequence similarities with luqin-type neuropeptides from molluscs and annelids, including a C-terminal RFamide motif and the N-terminal motif WRPQ, which is conserved with only conservative substitutions (Figure 1). Interestingly, only two of the four precursors have a C-terminal pair of cysteines separated by 10 amino acids, which is typically a conserved feature of luqin-type precursors in other phyla (Koziol et al., 2016). The functional significance of the loss of this feature in two precursors is unknown, but it may be reflective of a loss of selection pressure after gene duplications gave rise to the four precursor genes.

Genes encoding luqin-type precursors have also been identified in parasitic platyhelminths, including the fox tapeworm *Echinococcus multilocularis*, the salmon fluke *Gyrodactlyus salaris*, the rodent tapeworm *Hymenolepis microstoma*, the broad fish tapeworm *Diphyllobothrium latum*, and the cestode *Mesocestoides corti* (Koziol et al., 2016). In all these species, a single luqin-type precursor comprising a single predicted neuropeptide was identified, with the peptides having the N-terminal motif WRPH that is similar to the WRPQ motif that is a feature of luqin-type neuropeptides in molluscs and annelids. Interestingly, however, the luqin-type peptides in these species are positioned in the C-terminal region of the precursor protein. This contrasts with precursors in other taxa, where luqin-type peptides are located N-terminally and proximal to the signal peptide. Furthermore, none of the luqin-type precursors in the parasitic platyhelminth species listed above have a C-terminal pair of cysteines separated by 10 amino acids. Thus, the luqin-type precursors identified in parasitic platyhelminthes are highly divergent by comparison with other luqin-type precursors. This makes sequence alignment difficult and for this reason luqin-type precursors from parasitic platyhelminths are not included in Figure 1 but instead they are shown in Supplementary Figure 1B. Nevertheless, evidence that luqin-type neuropeptides in parasitic platyhelminths are functional can be found in the identification of genes encoding candidate luqin-type receptors; for example, in *E. multilocularis* (Koziol et al., 2016). Furthermore, a comprehensive analysis of GPCRs encoded in the genome of the parasitic helminth *Fasciola hepatica* has revealed the presence of a receptor that is clearly an ortholog of the luqin-type receptor from the annelid *P. dumerilii* and the RYamide receptor from *D. melanogaster* (McVeigh et al., 2018). Further studies are now needed to confirm the predicted luqin-type ligand-receptor partners in platyhelminths and to investigate the expression and pharmacological actions of luqin-type neuropeptides in platyhelminths.

Luqin-type precursors have also been identified in the brachiopod *Lingula anatina* and in the nemertean *Lineus longissimus* (bootlace worm) (De Oliveira et al., 2019). In both species, a single luqin-type precursor was identified that contains a predicted mature neuropeptide with the C-terminal sequence WRPQGRF-NH_2_, which is the same motif identified in molluscan and annelid luqin-type peptides. The precursors identified in these species also have the typical C-terminal region containing the two cysteines separated by 10 amino acid residues (Figure 1). Furthermore, two proteins in *L. anatina* have been annotated as luqin-type receptors (XP_013402794.1, XP_013402807.1).

## RYamides: Luqin-Type Neuropeptides in Arthropods

Arthropodan neuropeptides with a C-terminal RYamide motif (RYamides) were first identified in the decapod *C. borealis* by *de novo* post source decay sequencing of peptides in extracts of the pericardial organs of this species. Five different peptides were identified, all of them sharing the conserved C-terminal motif FXXXRY-NH_2_, where X is variable (Li et al., 2003). RYamides sharing the same C-terminal motif were subsequently identified by analysis of tissue extracts from other decapods, including *Cancer productus* (Fu et al., 2005), *Pugettia producta* (Stemmler et al., 2007), *Carcinus maenas* (Ma et al., 2009), and in the Pacific white shrimp *Litopenaeus vannamei* (Ma et al., 2010).

Genes/transcripts encoding precursors of RYamides have been identified in several crustacean species, including the water flea *Daphnia pulex* (Dircksen et al., 2011), the isopod *Proasellus cavaticus* (Christie, 2017), the red swamp crayfish *Procambarus clarkii* (Veenstra, 2015), the Australian crayfish *Cherax quadricarinatus* (Nguyen et al., 2016), and the freshwater amphipod *Hyalella azteca* (Christie et al., 2018). Interestingly, while most of these precursors comprise two RYamides, the *D. pulex* and *P. clarkii* precursors comprise three predicted RYamides (Supplementary Figure 1A). In the case of the *D. pulex* RYamide precursor, mass spectrometric analysis of brain tissue enabled identification of two of the three RYamides predicted to be derived from this precursor. The first peptide, located immediately after the signal peptide, was identified in its post-translationally modified form as pQTFFTNGRY-NH_2_, with both C-terminal amidation and N-terminal conversion of a glutamine residue to pyroglutamate. The second RYamide predicted to be derived from this precursor was not detected by mass spectrometry. Two different forms of the third RYamide peptide were detected—the 27 residue peptide SGNGGIVLGNSELDARNPERFFIGSRY-NH_2_ and a C-terminal fragment of this peptide (NPERFFIGSRY-NH_2_) generated by cleavage at the underlined arginine residue in the longer peptide (Dircksen et al., 2011).

Genes/transcripts encoding precursors of RYamides have also been identified in a variety of insects, including six *Drosophila* species, the red flour beetle *T. castaneum*, the silkworm *Bombyx mori*, the honey bee *Apis mellifera*, the pea aphid *Acyrthosiphon pisum*, the yellow fever mosquitoes *Aedes aegypti* and *Culex pipiens*, and the stick insect *Carausius morosus* (Hauser et al., 2010; Liessem et al., 2018), and these typically comprise two predicted RYamides. Interestingly, in the RYamide precursor of the parasitic wasp *Nasonia vitripennis* paracopy expansion has occurred to give rise to a precursor comprising seven predicted RYamide-type neuropeptides (Supplementary Figure 1A). However, only one of these peptides has thus far been characterized biochemically by mass spectrometry (Hauser et al., 2010).

The first arthropod RYamide receptors to be characterized were from *T. castaneum* and *D. melanogaster*. In the case of the red flour beetle *T. castaneum*, a receptor was identified (GenBank Accession Number: HQ709383) and shown to be activated in a dose-dependent manner by the two RYamide peptides derived from the *T. castaneum* RYamide precursor (Collin et al., 2011). In *D. melanogaster*, the RYamide receptor was characterized independently by two laboratories, revealing that the two RYamides derived from the *D. melanogaster* RYamide precursor activate, in a dose-dependent manner, a receptor encoded by the gene CG5811 (Collin et al., 2011; Ida et al., 2011). Ida et al. (2011) also reported that injection of RYamide-1 suppresses the proboscis extension reflex (PER) in the blowfly *Phormia regina*, indicating a physiological role in regulation of feeding behavior (Ida et al., 2011). Subsequent experimental studies on this species revealed the presence of 26 RYamide-immunoreactive neurons in the brain and showed that injection of RYamide-1 or -2 has no effect on the volume of sucrose solution intake when feeding occurs but causes a reduction in the percentage of flies exhibiting the PER. Furthermore, injection of RYamide-1 was found to cause a significant decrease in the responsiveness to sucrose solution of sugar receptor neurons located on the labellum of the proboscis. Thus, it was concluded that RYamides suppress feeding motivation and sucrose responsiveness in the blow fly *P. regina* (Maeda et al., 2015). Analysis of the expression of the RYamide precursor in the silkworm *B. mori* using mRNA *in situ* hybridization revealed expression in the brain, terminal abdominal ganglion, and midgut. In the larval and adult brain, four to seven pairs of RYamide precursor-expressing neurons were identified in the protocerebrum and tritocerebrum. Expression was also revealed in a pair of posterior dorsomedial neurons in the terminal abdominal ganglion of adults and larvae. Lastly, RYamide precursor expression was revealed in enteroendocrine cells of the anterior midgut of larvae, pupae, and adult specimens of *B. mori.* This pattern of expression indicates that RYamides are involved in regulation of feeding and digestion in *B. mori* (Roller et al., 2016).

Consistent with the hypothesis that RYamides may also regulate feeding behavior in other arthropods, use of quantitative real-time PCR revealed that expression of the RYamide precursor gene is significantly downregulated in the brain after starvation in the decapod *Marsupenaeus japonicus* (kuruma shrimp). Furthermore, injection of RYamides into the muscle of juvenile *M. japonicus* caused suppression of food intake in some experiments, but this was not consistently reproducible (Mekata et al., 2017). Foregut activity in decapods is controlled by motor neurons in the stomatogastric ganglion (STG), the activity of which is regulated by a variety of different neuropeptide types (Marder and Bucher, 2007). To gain further insights into the complexity of neuropeptide signaling in the STG, transcriptomic analysis of the STG in the crab *C. borealis* revealed the presence of 46 transcripts encoding receptors for 27 different neuropeptide types, but interestingly RYamide receptor transcripts were not detected. Consistent with this finding, *in vitro* application of RYamides to *C. borealis* STG preparations had no consistent modulatory effect on the motor outputs of the ganglion (Dickinson et al., 2019). Thus, further studies are now required to investigate the mechanisms by which RYamides may affect feeding in decapod crustaceans.

RYamides are not only involved in regulation of feeding behavior in arthropods. Analysis of RYamide expression in several *Drosophila* species revealed that the RYamide precursor gene is expressed in two abdominal neurons of the adult central nervous system that project to the rectal papillae, organs that mediate water re-absorption in flies (Veenstra and Khammassi, 2017). Consistent with this expression pattern, injection of female mosquitoes with RYamides delays postprandial diuresis (Veenstra and Khammassi, 2017). Thus, it appears that RYamides may act as regulators of urine production in insects. Interestingly, this is consistent with immunohistochemical evidence of a similar role in the mollusc *Aplysia*, where luqin-immunoreactivity has been localized in a nerve associated with muscles of the renal pore, a sphincter that controls urine efflux (Angers et al., 2000) (see above).

## Detailed Functional Characterization of Luqin-Type Signaling in the Nematode *Caenorhabditis elegans*

In 2003, Keating et al. reported an extensive functional analysis of GPCRs in the nematode *C. elegans*, employing use of RNA interference (RNAi) methods to knockdown GPCR gene expression. Included in this study was the gene *Y59H11AL.1* (also now known as *npr-22*) and a phylogenetic analysis revealed that the protein encoded by *Y59H11AL.1* is most closely related to the *L. stagnalis* luqin receptor GRL106. However, both the receptor encoded by *Y59H11AL.1* and GRL106 were classified by the authors under the general descriptor of “tachykinin-like receptors” (Keating et al., 2003). Subsequently, efforts were made to identify the neuropeptide(s) that act as a ligand for the *Y59H11AL.1*-encoded GPCR (NPR-22) by screening a library of synthetic neuropeptides predicted from analysis of the sequences of *C. elegans* neuropeptide precursors (Mertens et al., 2006). It was discovered that FMRFamide related peptides derived from the FLP-7 precursor can activate NPR-22, with the peptide FLP-7.3 (SPMERSAMVRF-NH_2_) being the most potent, albeit with an EC_50_ of ∼1 μM (Mertens et al., 2006). The authors also noted that NPR-22 (Y59H11AL.1) is closely related to the *D. melanogaster* receptor CG5811. However, at the time the peptides that act as ligands for CG5811 were unknown and it was not until 5 years later that it was discovered that CG5811 is a RYamide receptor (Collin et al., 2011; Ida et al., 2011).

In 2013, an extensive phylogenetic analysis of GPCRs and neuropeptide precursors identified a luqin-type precursor in *C. elegans* (Mirabeau and Joly, 2013). Furthermore, alignment of the *C. elegans* precursor with luqin-type precursors from other taxa revealed many conserved features. Thus, the precursor comprises two putative luqin-type peptides that have an RYamide motif and the C-terminal region of the precursor contains two cysteines, which are separated by eight amino acid residues (Mirabeau and Joly, 2013). The spacing of the two cysteines residues in the *C. elegans* luqin-type precursor is atypical of luqin-type precursors where, as highlighted above and shown in Figure 1, the two cysteine residues are usually separated by ten residues. However, this feature of the *C. elegans* luqin-type precursor is probably a derived characteristic because in another nematode species, the parasite *Trichuris suis*, the luqin-type precursor has two C-terminal cysteines separated by 10 residues (Figure 1) (Yañez-Guerra et al., 2018).

**FIGURE 2:**
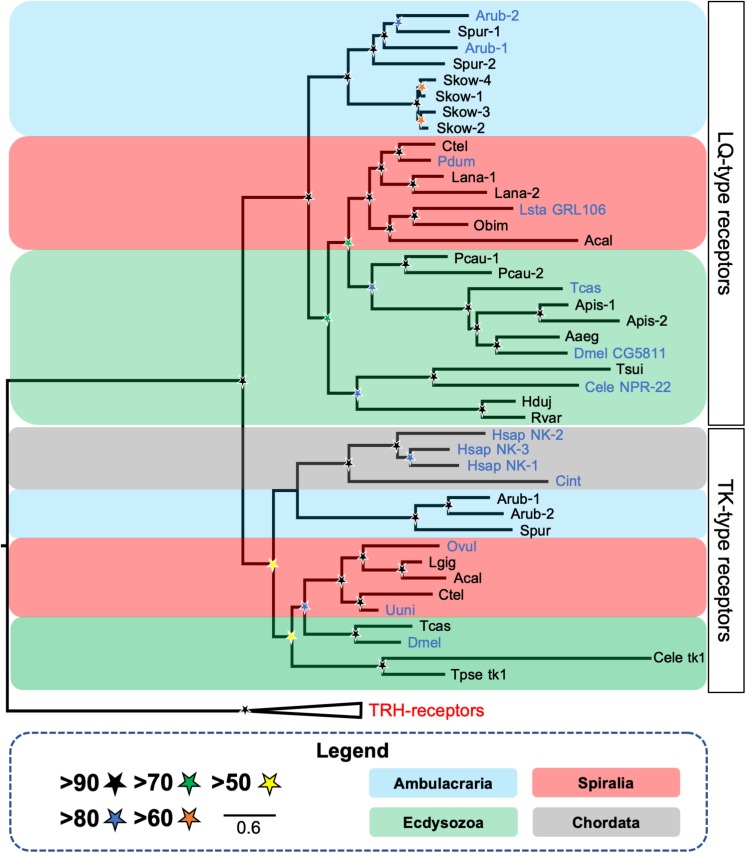
Phylogenetic tree showing the occurrence and relationships of luqin/RYamide-type receptors and tachykinin-type receptors in bilaterians. The tree comprises two distinct receptor clades—luqin/RYamide-type receptors and the paralogous tachykinin-type receptors, with thyrotropin-releasing hormone (TRH)-type receptors included as an outgroup. Taxa are color-coded and SH-aLRT support (Guindon et al., 2010) [1000 replicates] for clades is represented with colored stars, as explained in the key. Species in which the peptide ligands that activate luqin/RYamide-type receptors or tachykinin-type receptors have been identified experimentally are shown with blue lettering. Species names are as follows: Aaeg (*Aedes aegypti*), Acal (*Aplysia californica*), Apis (*Acyrthosiphon pisum*), Arub (*Asterias rubens*), Cele (*Caenorhabditis elegans*), Cint (*Ciona intestinalis*), Ctel (*Capitella teleta*), Dmel (*Drosophila melanogaster*), Hduj (*Hypsibius dujardini*), Hsap (*Homo sapiens*), Lana (*Lingula anatina*), Lsta (*Lymnaea stagnalis*), Obim (*Octopus bimaculoides*), Ovul (*Octopus vulgaris*), Pcau (*Priapulus caudatus*), Pdum (*Platynereis dumerilii*), Rvar (*Ramazzottius varieornatus)*, Skow (*Saccoglossus kowalevskii*), Spur (*Strongylocentrotus purpuratus*), Tcas (*Tribolium castaneum*), Tsui (*Trichuris suis*), Tpse (*Trichinella pseudospiralis*), Uuni (*Urechis unicinctus*). This figure is a modified version of a tree reported previously in Yañez-Guerra et al. (2018), with the addition of published luqin-type receptor sequences from the tardigrades *H. dujardini* and *R. varieornatus* (Koziol, 2018) and the brachiopod *L. anatina* (XP_013402794.1, XP_013402807.1) and tachykinin-type receptor sequences from the nematodes *C. elegans* (tk-1; C38C10.1) (Mirabeau and Joly, 2013) and *T. spiralis* (KRY8989.1). The alignment was performed using MUSCLE (Edgar, 2004) [16 iterations] and the trimming was made using BMGE (Criscuolo and Gribaldo, 2010) [standard automatic trimming]. The tree was generated in W-IQ-tree (Trifinopoulos et al., 2016) using the maximum likelihood method with automatic selection of the substitution model. The branch support analysis used was SH-aLRT (Guindon et al., 2010) with 1000 iterations.

Recently, a detailed analysis of the phylogenetic relationships of luqin-type receptors revealed that the *C. elegans* receptor NPR-22 (Y59H11AL.1) is an ortholog of the *L. stagnalis* luqin receptor (GRL106) and the *D. melanogaster* receptor CG5811 and belongs to a clade of luqin-type receptors that is quite distinct from the closely related tachykinin-type receptors and neuropeptide-Y-type receptors (Yañez-Guerra et al., 2018) (Figure 2). Furthermore, the neuropeptides LURY-1.1 (PALLSRY-NH_2_) and LURY-1.2 (AVLPRY-NH_2_) derived from the *C. elegans* luqin/RYamide precursor (LURY-1) were shown to act as ligands for NPR-22 at nanomolar concentrations (Ohno et al., 2017). Additionally, the authors showed that, as reported previously by Mertens et al. (2006), the FLP-7.3 peptide (SPMERSAMVRF-NH_2_) derived from the FLP-7 precursor also acts as a ligand for NPR-22, but only at micromolar concentrations (Ohno et al., 2017). Therefore, it is likely that LURY-1.1 and LURY-1.2 are the natural ligands for NPR-22 and the ability of FLP-7 derived-peptides to activate NPR-22 at micromolar concentrations may be an *in vitro* pharmacological and non-physiological phenomenon due to the presence of a C-terminal RYamide-like RFamide motif in these peptides. However, evidence that NPR-22 mediates effects of FLP-7 derived peptides *in vivo* has been reported in an investigation of neuroendocrine mechanisms of serotonin-induced fat loss in *C. elegans* (Palamiuc et al., 2017).

Having identified the molecular components of a luqin/RYamide-type neuropeptide signaling system in *C. elegans*, Ohno et al. (2017) performed a detailed functional characterization of this signaling system. Analysis of the expression of the LURY-1 precursor and NPR-22 in *C. elegans* revealed that LURY-1 is expressed by the pharyngeal neurons M1 and M2, which regulate feeding in *C. elegans*. Thus, the M1 neuron stimulates spitting behavior whereas the M2 neuron stimulates pharyngeal pumping (Bhatla et al., 2015). With a much wider pattern of expression, NPR-22 is expressed in head muscles, I2 pharyngeal neurons, feeding pacemaker MC neurons, the RIH neuron, the interneurons AIA and AUA, the ASK neurons, the ASI neurons, a few B-type motoneurons in the posterior ventral nerve cord, pharyngeal muscles, body wall muscles, the intestine, and a few unidentified cells anterior to the nerve ring (Ohno et al., 2017; Palamiuc et al., 2017).

To gain insights into the physiological roles of LURY-1-derived peptides, the *lury-1* gene was overexpressed in *C. elegans*, with several phenotypes being observed. First, there was an increase in the number of unlaid eggs in the uterus but the rate of egg-laying was not affected, indicating that the rate of ovulation is normal but egg-laying is facilitated and embryos are laid prematurely. Accordingly, microinjection of synthetic LURY-1 peptides (10 μM) caused the same phenotype. Second, pharyngeal pumping, which is required for food intake, was reduced and microinjection of synthetic LURY-1 peptides (10 μM) caused the same phenotype. Third, adult lifespan was extended by as much as 21–50%. Finally, a reduction in locomotor activity was observed (Ohno et al., 2017). Importantly, all of these phenotypes were largely suppressed by the deletion of *npr-22*, indicating that LURY-1 peptides act upstream of NPR-22. Furthermore, these findings were consistent with a previous analysis of *npr-22* knockdown, which revealed a phenotype in which animals have a reduced body size and brood size (Ceron et al., 2007). More specifically, cell-specific rescue experiments indicated that NPR-22 acts in the feeding pacemaker MC neurons to control feeding and lifespan and NPR-22 acts upstream of the serotonin-uptaking RIH neuron to control egg-laying (Ohno et al., 2017). The authors conclude that food-evoked activation of the pharynx triggers MC neurons to release of LURY-1 peptides, which then act as hormones via NPR-22-dependent mechanisms to control feeding, egg-laying, and roaming behavior (Ohno et al., 2017). Thus, use of *C. elegans* as a model experimental system has provided the first whole-animal perspective on the physiological/behavioral roles of luqin-type neuropeptide signaling.

## RYamide-Type Neuropeptide Precursors in Other Ecdysozoans

Analysis of transcriptome sequence data from the penis worm *Priapulus caudatus* (phylum Priapulida) revealed the existence of a luqin-type neuropeptide precursor in this species (Yañez-Guerra et al., 2018). This precursor comprises two putative luqin-type neuropeptides with a C-terminal RYamide motif and has the conserved C-terminal region with two cysteines separated by 10 amino acid residues (Figure 1). Interestingly, the *P. caudatus* neuropeptides share similarities with both arthropod/nematode RYamides and spiralian luqins. Thus, while the C-terminal RYamide motif is a conserved feature of this neuropeptide family in ecdysozoans, the N-terminal region of the *P. caudatus* luqin-type neuropeptides shares more sequence similarity with mollusc/annelid luqins than with arthropod/nematode RYamides. For example, the N-terminal sequence QWRP in one of the *P. caudatus* RYamides is also a feature of several molluscan luqin-type peptides (Figure 1). These “intermediate” characteristics of the *P. caudatus* RYamides are also reflected in a phylogenetic analysis of the relationships of luqin/RYamide-type precursors, where the *P. caudatus* RYamide precursor is not positioned in a clade comprising arthropod/nematode precursors, as would be expected based on animal phylogenetic relationships, but instead it is positioned at the base of a clade comprising mollusc/annelid precursors. This suggests that the *P. caudatus* RYamide precursor may have retained many of the ancestral characteristics of protostome luqin-type precursors, whereas the arthropod/nematode luqin-type precursors appear to be more divergent (Yañez-Guerra et al., 2018). In *P. caudatus*, two candidate receptors for luqin-type neuropeptides have been identified based on their phylogenetic relationship with luqin-type receptors that have been characterized in other protostomes (Figure 2) (Yañez-Guerra et al., 2018). Experimental studies are now needed to determine if the two *P. caudatus* luqin-type neuropeptides are effective as ligands for both receptors or if the receptors exhibit preferential ligand binding. Furthermore, experimental studies are needed to investigate the physiological roles of luqin-type signaling in priapulids.

Analysis of the genome sequences of the tardigrades *Hypsibius dujardini* and *Ramazzottius varieornatus* (phylum Tardigrada) has revealed the occurrence of luqin-type precursors and receptors in both of these species (Koziol, 2018). One luqin-type precursor was identified in *H. dujardini* and two luqin-type precursors were identified in *R. varieornatus* (Koziol, 2018). The predicted neuropeptides derived from these precursors have a C-terminal RYamide motif, consistent with other members of this neuropeptide family in ecdysozoans. However, atypical of ecdysozoan luqin-type precursors, the tardigrade precursors comprise only one predicted neuropeptide (Figure 1). The C-terminal region of the precursor contains two cysteines separated by 10 amino acid residues in *H. dujardini* and in one of the two precursors from *R. varieornatus*, while the second precursor in *R. varieornatus* lacks the second cysteine (Figure 1). Genes encoding a single luqin-type receptor have been identified in both *H. dujardini* and *R. varieornatus* (Koziol, 2018) but the ligand-binding properties of these receptors remain to be investigated experimentally. Furthermore, nothing is known about the physiological roles of luqin-type neuropeptides in tardigrades and so this will be an interesting area for investigation in the future, particularly in the context of their remarkable capacity to withstand extreme environmental conditions, including radiation tolerance, desiccation, and both high and low temperature and pressure (Jönsson, 2019; Jönsson et al., 2019; Kamilari et al., 2019).

## Discovery of Luqin-Type Signaling in Ambulacrarian Deuterostomes Reveals the Urbilaterian Origin of This Neuropeptide Signaling System

The discovery of precursors of luqin-type peptides in deuterostomian invertebrates was first reported in 2013. They were identified in the hemichordate *Saccoglossus kowalevskii* and in the echinoderm *Strongylocentrotus purpuratus*, which was facilitated by the presence of the aforementioned conserved C-terminal region containing two cysteines separated by 10 amino acid residues (Jékely, 2013). The *S. purpuratus* and *S. kowalevskii* precursor proteins comprise the putative neuropeptides EIRSPGGKPHKFMRW-NH_2_ and EGSNTFLRW-NH_2_, respectively. Thus, the presence of a C-terminal FXRW-NH_2_ motif (where X is L or M) was identified as a characteristic feature of luqin-type peptides in the ambulacrarian clade of the deuterostomes (Elphick and Mirabeau, 2014).

Through analysis of transcriptome/genome sequence data, luqin-type neuropeptide precursors have subsequently been identified in other echinoderm classes, including Holothuroidea (sea cucumbers), Asteroidea (starfish or sea stars), and Ophiuroidea (brittle stars). In the sea cucumbers *Apostichopus japonicus*, *Holothuria glaberrima*, *Holothuria scabra*, and *Holothuria leucospilota*, the precursor comprises a single neuropeptide with the same predicted structure in all four species—KPYKFMRW-NH_2_ (Rowe et al., 2014; Suwansa-Ard et al., 2018; Chen et al., 2019; Chieu et al., 2019). Luqin-type precursors identified in the starfish species *A. rubens* and *Acanthaster planci* comprise a single putative neuropeptide with the amino acid sequence EKGRFPKFMRW-NH_2_ and EEKTRFPKFMRW-NH_2_, respectively (Semmens et al., 2016; Smith et al., 2017). Ophiuroid luqin-type precursors also comprise a single putative neuropeptide, which has the predicted sequence QGFNRDGPAKFMRW-NH_2_ in *Ophionotus victoriae*, QGFNRGEGPAKFMRW-NH_2_ in *Ophiopsila aranea*, and QGFSRDGPAKFMRW-NH_2_ in *Amphiura filiformis* (Zandawala et al., 2017). Thus, the C-terminal motif KFMRW-NH_2_ appears to be a conserved feature of luqin-type neuropeptides in echinoderms.

**FIGURE 3:**
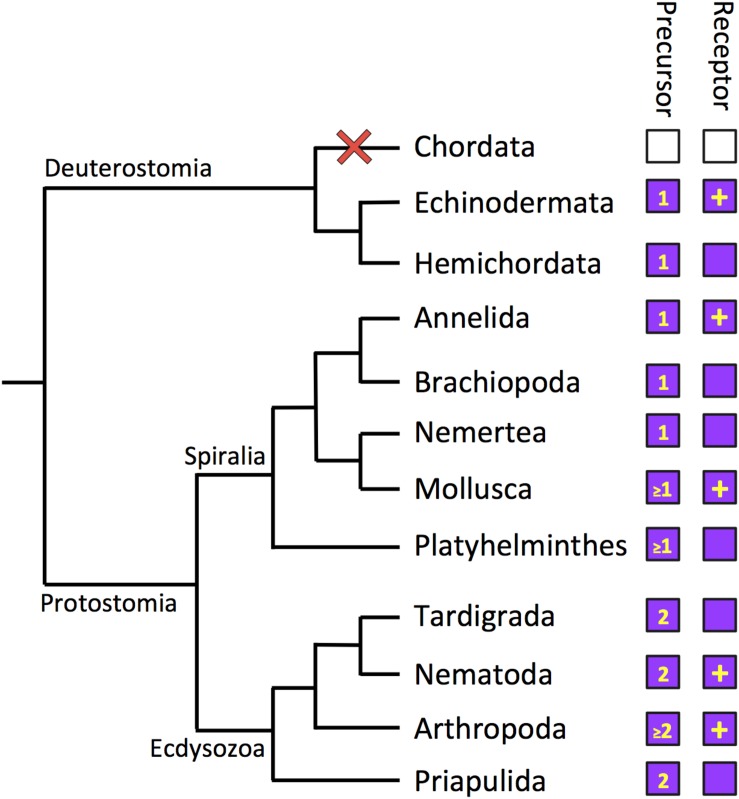
Phylogenetic diagram showing the occurrence of luqin-type neuropeptide signaling in the Bilateria. The phylogenetic tree shows relationships of selected bilaterian phyla. The phyla in which luqin-type precursors and luqin-type receptors have been identified are labeled with purple-filled boxes. The number in the precursor box indicates how many luqin-type neuropeptides are known or predicted to be derived from the precursor protein. The inclusion of a plus symbol in the receptor boxes indicates that the peptide ligand(s) that activates the receptor has been determined experimentally. Note the loss of the luqin-type signaling system in the chordate lineage, which is signified by the red cross and the white-filled boxes. Note that Xenacoelomorpha are not included in this diagram because of the controversy regarding the phylogenetic position of this phylum. However, as discussed in this review, luqin-type receptors have been identified in xenacoelomorphs but the precursors of peptides that act as ligands for these receptors have yet to be identified. The cladogram depicting bilaterian relationships is based on a recent phylogenetic study reported by Laumer et al. (2019).

A large-scale analysis of the phylogenetic distribution of G-protein coupled neuropeptide receptors in bilaterian animals revealed the presence of luqin-type receptors in ambulacrarians (hemichordates and echinoderms) (Mirabeau and Joly, 2013), consistent with the identification of luqin-type neuropeptide precursors in these taxa. However, luqin-type receptors were not identified in vertebrates or other chordates (urochordates and cephalochordates) and accordingly luqin-type neuropeptide precursors have not been identified in these taxa. Thus, it was concluded that the evolutionary origin of luqin-type receptors can be traced to the common ancestor of protostomes and deuterostomes, but with subsequent loss in the chordate lineage (Mirabeau and Joly, 2013). Furthermore, the phylogenetic analysis of neuropeptide receptor relationships reported by Mirabeau and Joly (2013) revealed that luqin-type receptors are paralogs of tachykinin-type receptors and this finding was confirmed recently by a phylogenetic analysis specifically focused on luqin-type receptors and closely related neuropeptide receptors (Yañez-Guerra et al., 2018) (Figure 2). Thus, it can be inferred that gene duplication in a common ancestor of the Bilateria gave rise to the paralogous luqin-type and tachykinin-type signaling systems, but with subsequent loss of luqin-type signaling in chordates (Figure 3).

A detailed analysis of luqin-type receptors in ambulacrarians revealed the presence of four genes/transcripts in the hemichordate *S. kowalevskii* and two genes/transcripts in the echinoderms *S. purpuratus* (sea urchin) and *A. rubens* (starfish) that encode members of this family of neuropeptide receptors (Yañez-Guerra et al., 2018). Furthermore, the starfish *A. rubens* was selected a model experimental system in which to functionally characterize luqin-type neuropeptide signaling for the first time in a deuterostome. A cDNA encoding the *A. rubens* luqin-type precursor ArLQP was cloned and the structure of the mature peptide derived from this precursor was determined using mass spectrometry as a 12 amino acid residue peptide that is C-terminally amidated—EEKTRFPKFMRW-NH_2_ (ArLQ). Cloning, sequencing, and heterologous expression of cDNAs encoding two *A. rubens* luqin-type receptors (ArLQR1 and ArLQR2) facilitated testing of synthetic ArLQ as a candidate ligand for these receptors. This revealed that ArLQ is a potent ligand for both ArLQR1 and ArLQR2, with EC_50_ values of 2.4 × 10^–8^ and 7.8 × 10^–10^ M, respectively (Yañez-Guerra et al., 2018).

To gain insights into the physiological roles of luqin-type neuropeptide signaling in *A. rubens*, mRNA *in situ* hybridization methods were employed to investigate the expression pattern of ArLQP in adult starfish. ArLQP-expressing cells were revealed in the central nervous system, including the circumoral nerve ring and the radial nerve cords. However, expression was limited to the ectoneural region, which contains sensory and interneurons, with no expression detected in motoneuron cell bodies located in the hyponeural region. ArLQP-expressing cells were also revealed in starfish locomotor organs—tube feet—specifically located in close proximity to the basal nerve ring in the disk region. Lastly, in the digestive system, ArLQP-expressing cells were revealed in the cardiac stomach and pyloric stomach (Yañez-Guerra et al., 2018). Efforts to generate antibodies to ArLQ were also made to enable immunohistochemical analysis of ArLQ expression in *A. rubens*, but these were unsuccessful. Nevertheless, informed by the pattern of ArLQP expression revealed by use of mRNA *in situ* hybridization, synthetic ArLQ was tested as a potential myoactive peptide on *in vitro* preparations of tube feet and cardiac stomach. No effects on cardiac stomach preparations were observed but, interestingly, ArLQ was found to cause dose-dependent relaxation of tube foot preparations (Yañez-Guerra et al., 2018). Furthermore, the relaxing effect of ArLQ was similar in potency and magnitude to SALMFamide-2 (S2), a neuropeptide that has been identified and functionally characterized previously as a myorelaxant in *A. rubens* (Elphick et al., 1991; Melarange and Elphick, 2003). Clearly, further studies are now needed to gain broader insights into the physiological roles of luqin-type neuropeptides in starfish and other echinoderms. Further investigation of the physiological roles of luqin-type neuropeptide signaling in echinoderms could also be extended beyond adult animals to the free-swimming larval stage of these animals. Detailed anatomical analyses of neuropeptide precursor gene expression in larvae of *A. rubens* and *S. purpuratus* have been reported recently (Mayorova et al., 2016; Wood et al., 2018) but these studies did not incorporate analysis of the expression of luqin-type precursors. Therefore, this is also an important objective for future work on luqin-type neuropeptide signaling in echinoderms.

## Luqin-Type Neuropeptide Signaling in Xenacoelomorphs: Receptors with Missing Ligands

The phylum Xenacoelomorpha comprises an assemblage of marine worms that have a simple body plan without a through-gut (Hejnol and Pang, 2016; Telford and Copley, 2016; Gavilán et al., 2019). They are of particular interest for evolutionary studies because of controversy regarding their phylogenetic position in the animal kingdom. On the one hand, they have been placed as a sister group to all other bilaterian animals [Nephrozoa hypothesis] (Cannon et al., 2016; Rouse et al., 2016). Alternatively, they are considered to be closely related to the Ambulacraria, forming a clade known as the Xenambulacraria [Xenambulacraria hypothesis] (Bourlat et al., 2006; Philippe et al., 2011). The most recent analysis of the phylogenetic position of xenacoelomorphs, including a strategy devoted to mitigate the effects of systematic errors, has supported the Xenambulacraria hypothesis (Philippe et al., 2019).

Analysis of transcriptome sequence data from 13 xenacoelomorph species revealed the occurrence of luqin-type receptors in this phylum (Thiel et al., 2018). Transcripts encoding luqin-type receptors were identified in species belonging to each of the three xenacoelomorph sub-phyla; Xenoturbellida (*Xenoturbella bocki*), Nemertodermatida (*Nemertoderma westbladi*), and Acoela (*Hofstenia miamia*). Furthermore, phylogenetic analysis revealed that these receptors form part of a clade of luqin-type receptors that include spiralian luqin receptors and ecdysozoan RYamide receptors. Interestingly, the xenacoelomorph luqin-type receptors are positioned within a branch that also includes ambulacrarian luqin-type receptors (Thiel et al., 2018). Therefore, this may be additional evidence in support of the Xenambulacraria hypothesis. Thus far, precursors of luqin-type neuropeptides have yet to be identified in xenacoelomorphs and so this represents an important objective for future work. In particular, it would be interesting to determine the C-terminal motif of luqin-type neuropeptides in xenacoelomorphs. If the peptides have a C-terminal RWamide motif, which is a characteristic of ambulacrarian luqin-type neuropeptides, then this would be further evidence of a close relationship between xenacoelomorphs and ambulacrarians. Furthermore, discovery of luqin-type neuropeptides in xenacoelomorphs would provide a basis for functional investigation of physiological roles of these neuropeptides in this phylum.

## General Conclusions and Speculations

The discovery and naming of luqin as a neuropeptide that is expressed in left upper quadrant cells of the abdominal ganglion of the mollusc *Aplysia* was perhaps a rather esoteric beginning to a new field of neuropeptide research. However, gradually over a period of more than three decades, it has become apparent that in fact this finding has broad relevance to neuropeptide signaling in bilaterian animals, with the notable exception of chordates. Although the name luqin was highly specific in its derivation, it nevertheless provides a useful generic name for the neuropeptide family. Thus, it is preferable to RFamides, RYamides, or RWamides because these C-terminal motifs are not unique to or even generally applicable to the neuropeptide family as whole. Therefore, our recommendation is that all members of this neuropeptide family are referred to as “luqins,” recognizing of course that the derivation of the name is meaningless beyond *Aplysia*.

Comparison of the sequences of luqin-type precursors has revealed variability in the number of luqin-type neuropeptides derived from these proteins. All the luqin-type precursors identified thus far in ambulacrarians comprise a single luqin-tye neuropeptide and this is also a feature of some luqin-type precursors in spiralians and ecdysozaons (Figure 1). Therefore, it could be inferred that this may reflect the characteristics of the luqin-type precursor in the common ancestor of the Bilateria. However, the existence of precursors that comprise two luqin-type neuropeptides is a feature of many ecdysozoans and some spiralian species, indicating perhaps that this characteristic has evolved independently in both lineages (Figure 1). Further expansions in the number of luqin-type neuropeptides derived from precursor proteins are seen in some crustacean species; for example, in *D. pulex* and *P. cavaticus*, where there are three predicted mature peptides (Dircksen et al., 2011; Christie, 2017) (Supplementary Figure 1A). However, the most extreme example is seen in the wasp *N. vitiprenis*, where the precursor comprises seven predicted luqin-type neuropeptides (Hauser et al., 2010) (Supplementary Figure 1A). The functional significance of this expansion in some arthropods is as yet unknown. Furthermore, the existence of multiple genes encoding luqin-type neuropeptides is a feature of some platyhelminth species (Koziol et al., 2016).

Thus far, genes/transcripts encoding luqin-type precursors and/or luqin-type receptors have been discovered in all of the non-chordate bilaterian phyla that have been investigated, but not in chordates (Figure 3). The loss of LQ-type signaling in chordates is of interest from a functional perspective, as discussed below. However, loss of LQ-type signaling in chordates has also influenced efforts to classify neuropeptide receptors. Hence, the original classification of the *C. elegans* and *L. stagnalis* LQ-type receptors as “tachykinin-like receptors” (Keating et al., 2003). More recently, in a phylogenetic analysis of gonadotropin-inhibitory hormone (GnIH)-type signaling, it was concluded that GnIH-type receptors have a “strong evolutionary relationship” with the *C. elegans* receptor NPR-22 (Ubuka and Tsutsui, 2018). This conclusion was a consequence of a phylogenetic analysis that was restricted to comparison of human GnIH-type receptors with a variety of *C. elegans* neuropeptide receptors, while more comprehensive phylogenetic analyses clearly show that the *C. elegans* receptor NPR-22 is an LQ-type receptor (Ohno et al., 2017; Yañez-Guerra et al., 2018).

While genes/transcripts encoding luqin-type precursors and/or luqin-type receptors have been discovered in all of the non-chordate bilaterian phyla that have been investigated, there are still many phyla that remain to be examined and so we cannot rule out the possibility that luqin-type neuropeptide signaling has been lost in some non-chordate phyla. Nevertheless, based on what it currently known, the loss of luqin signaling in chordates appears to be singular and therefore notable. Why then was luqin signaling lost in the chordates? To address this question, we need to consider, first, what is known about the physiological roles of luqin-type neuropeptide signaling and, second, the paralogous relationship of luqin-type signaling with tachykinin-type signaling.

**FIGURE 4:**
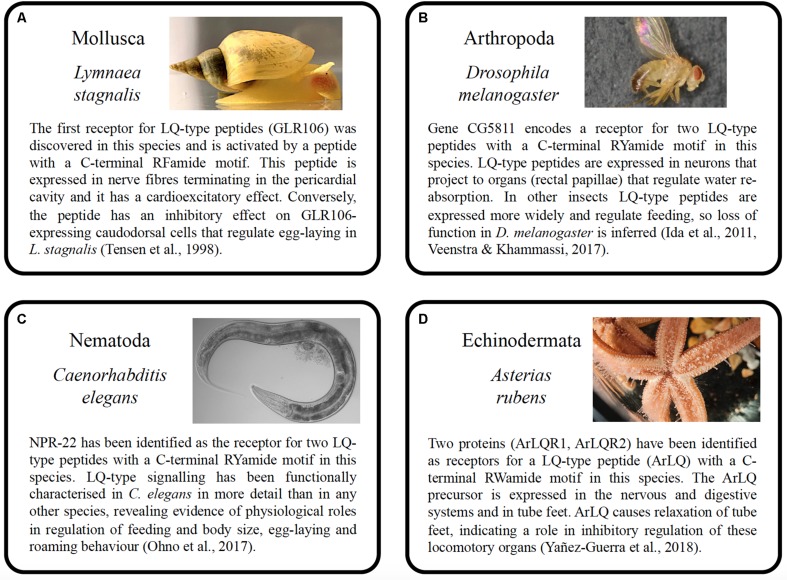
Summary of the properties and functions of luqin-type neuropeptide signaling in species belonging to four phyla. **(A)** Mollusca (*Lymnaea stagnalis*), **(B)** Arthropoda (*Drosophila melanogaster*), **(C)** Nematoda (*Caenorhabditis elegans*), and **(D)** Echinodermata (*Asterias rubens*). The photographs of the animals shown in **A**–**D** were taken by Michael Crossley (University of Sussex, United Kingdom), Marycruz Flores-Flores (Centro de Investigación y de Estudios Avanzados del Instituto Politécnico Nacional, Mexico), Marina Ezcurra (University of Kent, United Kingdom), and Ray Crundwell (Queen Mary University of London, United Kingdom).

Although luqin-type signaling was first discovered and then functionally characterized in molluscs, it was the recent detailed analysis of this signaling system in the nematode *C. elegans*, employing use of reverse genetic techniques, that has provided the most comprehensive insights into the physiological roles of luqin-type neuropeptide signaling. Furthermore, the findings from *C. elegans* provide a broad functional context for comparison with experimental findings from other less intensely studied taxa (Figure 4).

A key finding from *C. elegans* was that luqin-type neuropeptides are secreted by a pair of pharyngeal neurons (M1 and M2) and act as hormones to suppress feeding (Ohno et al., 2017). It is noteworthy, therefore, that luqin-type RYamides suppress feeding motivation and sucrose responsiveness in an insect (Maeda et al., 2015) and brain expression of the luqin-type RYamide precursor gene is significantly downregulated after starvation in a crustacean (Mekata et al., 2017). Thus, in ecdysozoans, there is evidence of an evolutionarily conserved role of luqin-type neuropeptide signaling as an inhibitory regulator of feeding in association with changes in food availability. Further studies are now required to investigate if luqin-type neuropeptides also regulate feeding activity in spiralians and ambulacrarians. Nevertheless, it is noteworthy that in the mollusc *A. fullica* luqin has excitatory effects on buccal neurons and muscles (Fujimoto et al., 1990) and in the starfish *A. rubens* a luqin-type neuropeptide precursor is expressed in the cardiac stomach, a region of the digestive system that is everted when starfish feed (Yañez-Guerra et al., 2018).

Luqin-type neuropeptides also regulate egg laying in *C. elegans*, acting via the luqin-type receptor NPR-22 upstream of the serotonin-uptaking RIH neuron. Thus, by comparison with wild-type animals, mutant worms lacking expression of the luqin-type LURY-1 precursor or NPR-22 exhibit reduced egg-laying during a period of refeeding after starvation (Ohno et al., 2017). Accordingly, detection of luqin-immunoreactivity in nerve fibers associated with the hermaphroditic ducts and the ovotestis and in nerve fibers associated with inhibition of the egg-laying hormone-producing caudodorsal cells in *L. stagnalis*, which express luqin-type receptor transcripts (Tensen et al., 1998), suggest that luqin-type signaling may likewise regulate egg laying in molluscs.

Overexpression of luqin-type neuropeptides in *C. elegans* produced a phenotype where worms exhibited reduced roaming activity and, importantly, this was not observed in animals lacking the luqin-type receptor NPR-22. It was concluded that this action may reflect a physiological role of luqin signaling in attenuating locomotory activity when food is abundant (Ohno et al., 2017). It is noteworthy, therefore, that the relaxing effect of the luqin-type neuropeptide ArLQ on starfish tube feet may likewise be consistent with a physiological role in inhibitory regulation of locomotor activity in an echinoderm (Yañez-Guerra et al., 2018). Investigation of the effects of ArLQ on locomotor activity will therefore be an interesting objective for future studies, employing use of methods that have been reported recently to examine the effects of other neuropeptides on starfish locomotor activity (Tinoco et al., 2018).

The discovery that luqin-type signaling is paralogous to tachykinin-type signaling was based on phylogenetic analysis of the relationships of G-protein coupled neuropeptide receptors in the Bilateria (Mirabeau and Joly, 2013; Thiel et al., 2018; Yañez-Guerra et al., 2018) (Figure 3). Therefore, it can be inferred that duplication of genes encoding a common ancestral neuropeptide precursor and receptor occurred in a common ancestor of the Bilateria to give rise to the paralogous luqin-type and tachykinin-type signaling systems. Analysis of the phylogenetic distribution of tachykinin-type signaling indicates that it has been retained in all bilaterian phyla that have been analyzed (Mirabeau and Joly, 2013; Elphick et al., 2018).

A possible explanation for the loss of a neuropeptide signaling system in a taxon could be functional redundancy with respect to a paralogous signaling system. However, given (i) the length of time elapsed since the gene duplications that gave rise to the paralogous luqin-type and tachykinin-type signaling systems is likely to be in excess of 650 million years, based on the estimated time of divergence of protostomes and deuterostomes (Erwin et al., 2011) and (ii) the preservation of both signaling systems in most bilaterian phyla that have been analyzed, it seems unlikely that this explains the loss of luqin signaling in chordates. Further insights may be obtained as part of a broader investigation of the functional significance of the loss of bilaterian neuropeptide signaling systems. Examples of other neuropeptide types that have been lost in chordates include the leucokinin-type and pigment dispersing factor (PDF)-type signaling systems (Mirabeau and Joly, 2013) while more specifically corazonin-type signaling has been lost in vertebrates and urochordates but not in cephalochordates (Tian et al., 2016; Zandawala et al., 2018). Comparisons with these neuropeptide systems may therefore be informative in future efforts to draw general conclusions on the evolutionary and functional significance of neuropeptide loss in chordates.

## Author Contributions

Both authors contributed equally to the planning and writing of the review article. LY-G produced the figures.

## Conflict of Interest

The authors declare that the research was conducted in the absence of any commercial or financial relationships that could be construed as a potential conflict of interest.

## References

[B1] Aloyz R. S. DesGroseillers L. (1995). Processing of the L5-67 precursor peptide and characterization of LUQIN in the LUQ neurons of *Aplysia californica*. *Peptides* 16 331–338. 10.1016/0196-9781(94)00140-5 7784264

[B2] Angers A. DesGroseillers L. (1998). Alternative splicing and genomic organization of the L5-67 gene of *Aplysia californica*. *Gene* 208 271–277. 10.1016/s0378-1119(98)00009-2 9524280

[B3] Angers A. Zappulla J. P. Zollinger M. DesGroseillers L. (2000). Gene products from LUQ neurons in the abdominal ganglion are present at the renal pore of *Aplysia californica*. *Comp. Biochem. Physiol. B Biochem. Mol. Biol.* 126 435–443. 10.1016/s0305-0491(00)00217-0 11007186

[B4] Bauknecht P. Jékely G. (2015). Large-scale combinatorial deorphanization of Platynereis neuropeptide GPCRs. *Cell Rep.* 12 684–693. 10.1016/j.celrep.2015.06.052 26190115

[B5] Bhatla N. Droste R. Sando S. R. Huang A. Horvitz H. R. (2015). Distinct neural circuits control rhythm inhibition and spitting by the myogenic pharynx of *C. elegans*. *Curr. Biol.* 25 2075–2089. 10.1016/j.cub.2015.06.052 26212880 PMC4546535

[B6] Bourlat S. J. Juliusdottir T. Lowe C. J. Freeman R. Aronowicz J. Kirschner M. (2006). Deuterostome phylogeny reveals monophyletic chordates and the new phylum Xenoturbellida. *Nature* 444 85–88. 10.1038/nature05241 17051155

[B7] Brown R. O. Gusman D. Basbaum A. I. Mayeri E. (1985). Identification of *Aplysia* neurons containing immunoreactive FMRFamide. *Neuropeptides* 6 517–526. 10.1016/0143-4179(85)90113-1 4080111

[B8] Cannon J. T. Vellutini B. C. Smith J. Ronquist F. Jondelius U. Hejnol A. (2016). Xenacoelomorpha is the sister group to Nephrozoa. *Nature* 530 89–93. 10.1038/nature16520 26842059

[B9] Ceron J. Rual J.-F. Chandra A. Dupuy D. Vidal M. van den Heuvel S. (2007). Large-scale RNAi screens identify novel genes that interact with the *C. elegans* retinoblastoma pathway as well as splicing-related components with synMuv B activity. *BMC Dev. Biol.* 7:30. 10.1186/1471-213X-7-30 17417969 PMC1863419

[B10] Chen M. Talarovicova A. Zheng Y. Storey K. B. Elphick M. R. (2019). Neuropeptide precursors and neuropeptides in the sea cucumber *Apostichopus japonicus*: a genomic, transcriptomic and proteomic analysis. *Sci. Rep.* 9:8829. 10.1038/s41598-019-45271-3 31222106 PMC6586643

[B11] Chieu H. D. Suwansa-Ard S. Wang T. Elizur A. Cummins S. F. (2019). Identification of neuropeptides in the sea cucumber *Holothuria leucospilota*. *Gen. Comp. Endocrinol.* 283:113229. 10.1016/j.ygcen.2019.113229 31348958

[B12] Christie A. E. (2017). Neuropeptide discovery in *Proasellus cavaticus*: prediction of the first large-scale peptidome for a member of the Isopoda using a publicly accessible transcriptome. *Peptides* 97 29–45. 10.1016/j.peptides.2017.09.003 28893643

[B13] Christie A. E. Cieslak M. C. Roncalli V. Lenz P. H. Major K. M. Poynton H. C. (2018). Prediction of a peptidome for the ecotoxicological model *Hyalella azteca* (Crustacea; Amphipoda) using a de novo assembled transcriptome. *Mar. Genomics* 38 67–88. 10.1016/j.margen.2017.12.003 29395622

[B14] Collin C. Hauser F. Krogh-Meyer P. Hansen K. K. Gonzalez de Valdivia E. Williamson M. (2011). Identification of the *Drosophila* and *Tribolium* receptors for the recently discovered insect RYamide neuropeptides. *Biochem. Biophys. Res. Commun.* 412 578–583. 10.1016/j.bbrc.2011.07.131 21843505

[B15] Conzelmann M. Williams E. A. Krug K. Franz-Wachtel M. Macek B. Jékely G. (2013). The neuropeptide complement of the marine annelid *Platynereis dumerilii*. *BMC Genomics* 14:906. 10.1186/1471-2164-14-906 24359412 PMC3890597

[B16] Criscuolo A. Gribaldo S. (2010). BMGE (Block Mapping and Gathering with Entropy): a new software for selection of phylogenetic informative regions from multiple sequence alignments. *BMC Evol. Biol.* 10:210. 10.1186/1471-2148-10-210 20626897 PMC3017758

[B17] De Oliveira A. L. Calcino A. Wanninger A. (2019). Extensive conservation of the proneuropeptide and peptide prohormone complement in mollusks. *Sci. Rep.* 9:4846. 10.1038/s41598-019-40949-0 30890731 PMC6425005

[B18] Dickinson P. S. Hull J. J. Miller A. Oleisky E. R. Christie A. E. (2019). To what extent may peptide receptor gene diversity/complement contribute to functional flexibility in a simple pattern-generating neural network? *Comp. Biochem. Physiol. Part D Genomics Proteomics* 30 262–282. 10.1016/j.cbd.2019.03.002 30974344 PMC7080212

[B19] Dircksen H. Neupert S. Predel R. Verleyen P. Huybrechts J. Strauss J. (2011). Genomics, transcriptomics, and peptidomics of *Daphnia pulex* neuropeptides and protein hormones. *J. Proteome Res.* 10 4478–4504. 10.1021/pr200284e 21830762

[B20] Edgar R. C. (2004). MUSCLE: multiple sequence alignment with high accuracy and high throughput. *Nucleic Acids Res.* 32 1792–1797. 10.1093/nar/gkh340 15034147 PMC390337

[B21] Elphick M. R. Mirabeau O. (2014). The evolution and variety of RFamide-type neuropeptides: insights from deuterostomian invertebrates. *Front. Endocrinol.* 5:93. 10.3389/fendo.2014.00093 24994999 PMC4062910

[B22] Elphick M. R. Mirabeau O. Larhammar D. (2018). Evolution of neuropeptide signalling systems. *J. Exp. Biol.* 221(Pt 3):jeb151092. 10.1242/jeb.151092 29440283 PMC5818035

[B23] Elphick M. R. Price D. A. Lee T. D. Thorndyke M. C. (1991). The SALMFamides: a new family of neuropeptides isolated from an echinoderm. *Proc. Biol. Sci.* 243 121–127. 10.1098/rspb.1991.0020 1676515

[B24] Erwin D. H. Laflamme M. Tweedt S. M. Sperling E. A. Pisani D. Peterson K. J. (2011). The Cambrian conundrum: early divergence and later ecological success in the early history of animals. *Science* 334 1091–1097. 10.1126/science.1206375 22116879

[B25] Fu Q. Kutz K. K. Schmidt J. J. Hsu Y.-W. A. Messinger D. I. Cain S. D. (2005). Hormone complement of the *Cancer productus* sinus gland and pericardial organ: an anatomical and mass spectrometric investigation. *J. Comp. Neurol.* 493 607–626. 10.1002/cne.20773 16304631

[B26] Fujimoto K. Ohta N. Yoshida M. Kubota I. Muneoka Y. Kobayashi M. (1990). A novel cardio-excitatory peptide isolated from the atria of the African giant snail, *Achatina fulica*. *Biochem. Biophys. Res. Commun.* 167 777–783. 10.1016/0006-291x(90)92093-f 2322251

[B27] Gavilán B. Sprecher S. G. Hartenstein V. Martinez P. (2019). The digestive system of xenacoelomorphs. *Cell Tissue Res.* 377 369–382. 10.1007/s00441-019-03038-2 31093756

[B28] Giardino N. D. Aloyz R. S. Zollinger M. Miller M. W. DesGroseillers L. (1996). L5-67 and LUQ-1 peptide precursors of *Aplysia californica*: distribution and localization of immunoreactivity in the central nervous system and in peripheral tissues. *J. Comp. Neurol.* 374 230–245. 10.1002/(sici)1096-9861(19961014)374:2<230::aid-cne6>3.0.co;2-3 8906496

[B29] Guindon S. Dufayard J.-F. Lefort V. Anisimova M. Hordijk W. Gascuel O. (2010). New algorithms and methods to estimate maximum-likelihood phylogenies: assessing the performance of PhyML 3.0. *Syst. Biol.* 59 307–321. 10.1093/sysbio/syq010 20525638

[B30] Hauser F. Neupert S. Williamson M. Predel R. Tanaka Y. Grimmelikhuijzen C. J. P. (2010). Genomics and peptidomics of neuropeptides and protein hormones present in the parasitic wasp *Nasonia vitripennis*. *J. Proteome Res.* 9 5296–5310. 10.1021/pr100570j 20695486

[B31] Hejnol A. Pang K. (2016). Xenacoelomorpha’s significance for understanding bilaterian evolution. *Curr. Opin. Genet. Dev.* 39 48–54. 10.1016/j.gde.2016.05.019 27322587

[B32] Ida T. Takahashi T. Tominaga H. Sato T. Kume K. Ozaki M. (2011). Identification of the novel bioactive peptides dRYamide-1 and dRYamide-2, ligands for a neuropeptide Y-like receptor in *Drosophila*. *Biochem. Biophys. Res. Commun.* 410 872–877. 10.1016/j.bbrc.2011.06.081 21704020

[B33] Jékely G. (2013). Global view of the evolution and diversity of metazoan neuropeptide signaling. *Proc. Natl. Acad. Sci. U.S.A.* 110 8702–8707. 10.1073/pnas.1221833110 23637342 PMC3666674

[B34] Jékely G. Melzer S. Beets I. Kadow I. C. G. Koene J. Haddad S. (2018). The long and the short of it - a perspective on peptidergic regulation of circuits and behaviour. *J. Exp. Biol.* 221(Pt 3):jeb166710. 10.1242/jeb.166710 29439060

[B35] Jönsson K. I. (2019). Radiation tolerance in tardigrades: current knowledge and potential applications in medicine. *Cancers* 11:1333. 10.3390/cancers11091333 31505739 PMC6770827

[B36] Jönsson K. I. Holm I. Tassidis H. (2019). Cell biology of the tardigrades: current knowledge and perspectives. *Results Probl. Cell Differ.* 68 231–249. 10.1007/978-3-030-23459-1_10 31598859

[B37] Kamilari M. Jørgensen A. Schiøtt M. Møbjerg N. (2019). Comparative transcriptomics suggest unique molecular adaptations within tardigrade lineages. *BMC Genomics* 20:607. 10.1186/s12864-019-5912-x 31340759 PMC6652013

[B38] Keating C. D. Kriek N. Daniels M. Ashcroft N. R. Hopper N. A. Siney E. J. (2003). Whole-genome analysis of 60 G protein-coupled receptors in *Caenorhabditis elegans* by gene knockout with RNAi. *Curr. Biol.* 13 1715–1720. 10.1016/j.cub.2003.09.003 14521838

[B39] Koziol U. (2018). Precursors of neuropeptides and peptide hormones in the genomes of tardigrades. *Gen. Comp. Endocrinol.* 267 116–127. 10.1016/j.ygcen.2018.06.012 29935140

[B40] Koziol U. Koziol M. Preza M. Costábile A. Brehm K. Castillo E. (2016). De novo discovery of neuropeptides in the genomes of parasitic flatworms using a novel comparative approach. *Int. J. Parasitol.* 46 709–721. 10.1016/j.ijpara.2016.05.007 27388856

[B41] Laumer C. E. Fernández R. Lemer S. Combosch D. Kocot K. M. Riesgo A. (2019). Revisiting metazoan phylogeny with genomic sampling of all phyla. *Proc. Biol. Sci.* 286:20190831. 10.1098/rspb.2019.0831 31288696 PMC6650721

[B42] Li L. Kelley W. P. Billimoria C. P. Christie A. E. Pulver S. R. Sweedler J. V. (2003). Mass spectrometric investigation of the neuropeptide complement and release in the pericardial organs of the crab, *Cancer borealis*. *J. Neurochem.* 87 642–656. 10.1046/j.1471-4159.2003.02031.x 14535947

[B43] Li L. Moroz T. P. Garden R. W. Floyd P. D. Weiss K. R. Sweedler J. V. (1998). Mass spectrometric survey of interganglionically transported peptides in *Aplysia*. *Peptides* 19 1425–1433. 10.1016/s0196-9781(98)00094-1 9809658

[B44] Liessem S. Ragionieri L. Neupert S. Büschges A. Predel R. (2018). Transcriptomic and neuropeptidomic analysis of the stick insect, *Carausius morosus*. *J. Proteome Res.* 17 2192–2204. 10.1021/acs.jproteome.8b00155 29701990

[B45] Ma M. Bors E. K. Dickinson E. S. Kwiatkowski M. A. Sousa G. L. Henry R. P. (2009). Characterization of the *Carcinus maenas* neuropeptidome by mass spectrometry and functional genomics. *Gen. Comp. Endocrinol.* 161 320–334. 10.1016/j.ygcen.2009.01.015 19523386 PMC2888039

[B46] Ma M. Gard A. L. Xiang F. Wang J. Davoodian N. Lenz P. H. (2010). Combining *in silico* transcriptome mining and biological mass spectrometry for neuropeptide discovery in the Pacific white shrimp *Litopenaeus vannamei*. *Peptides* 31 27–43. 10.1016/j.peptides.2009.10.007 19852991 PMC2815327

[B47] Maeda T. Nakamura Y. Shiotani H. Hojo M. K. Yoshii T. Ida T. (2015). Suppressive effects of dRYamides on feeding behavior of the blowfly, *Phormia regina*. *Zool. Lett.* 1:35. 10.1186/s40851-015-0034-z 26649188 PMC4672552

[B48] Marder E. Bucher D. (2007). Understanding circuit dynamics using the stomatogastric nervous system of lobsters and crabs. *Annu. Rev. Physiol.* 69 291–316. 10.1146/annurev.physiol.69.031905.161516 17009928

[B49] Mayorova T. D. Tian S. Cai W. Semmens D. C. Odekunle E. A. Zandawala M. (2016). Localization of neuropeptide gene expression in larvae of an echinoderm, the starfish *Asterias rubens*. *Front. Neurosci.* 10:553. 10.3389/fnins.2016.00553 27990106 PMC5130983

[B50] McVeigh P. McCammick E. McCusker P. Wells D. Hodgkinson J. Paterson S. (2018). Profiling G protein-coupled receptors of *Fasciola hepatica* identifies orphan rhodopsins unique to phylum Platyhelminthes. *Int. J. Parasitol. Drugs Drug Resist.* 8 87–103. 10.1016/j.ijpddr.2018.01.001 29474932 PMC6114109

[B51] Mekata T. Kono T. Satoh J. Yoshida M. Mori K. Sato T. (2017). Purification and characterization of bioactive peptides RYamide and CCHamide in the kuruma shrimp *Marsupenaeus japonicus*. *Gen. Comp. Endocrinol.* 246 321–330. 10.1016/j.ygcen.2017.01.008 28062303

[B52] Melarange R. Elphick M. R. (2003). Comparative analysis of nitric oxide and SALMFamide neuropeptides as general muscle relaxants in starfish. *J. Exp. Biol.* 206 893–899. 10.1242/jeb.00197 12547944

[B53] Mertens I. Clinckspoor I. Janssen T. Nachman R. Schoofs L. (2006). FMRFamide related peptide ligands activate the *Caenorhabditis elegans* orphan GPCR Y59H11AL.1. *Peptides* 27 1291–1296. 10.1016/j.peptides.2005.11.017 16377032

[B54] Mirabeau O. Joly J.-S. (2013). Molecular evolution of peptidergic signaling systems in bilaterians. *Proc. Natl. Acad. Sci. U.S.A.* 110 E2028–E2037. 10.1073/pnas.1219956110 23671109 PMC3670399

[B55] Nguyen T. V. Cummins S. F. Elizur A. Ventura T. (2016). Transcriptomic characterization and curation of candidate neuropeptides regulating reproduction in the eyestalk ganglia of the Australian crayfish, *Cherax quadricarinatus*. *Sci. Rep.* 6:38658. 10.1038/srep38658 27924858 PMC5141488

[B56] Ohno H. Yoshida M. Sato T. Kato J. Miyazato M. Kojima M. (2017). Luqin-like RYamide peptides regulate food-evoked responses in *C. elegans*. *eLife* 6:e28877. 10.7554/eLife.28877 28847365 PMC5576490

[B57] Palamiuc L. Noble T. Witham E. Ratanpal H. Vaughan M. Srinivasan S. (2017). A tachykinin-like neuroendocrine signalling axis couples central serotonin action and nutrient sensing with peripheral lipid metabolism. *Nat. Commun.* 8:14237. 10.1038/ncomms14237 28128367 PMC5290170

[B58] Philippe H. Brinkmann H. Copley R. R. Moroz L. L. Nakano H. Poustka A. J. (2011). Acoelomorph flatworms are deuterostomes related to *Xenoturbella*. *Nature* 470 255–258. 10.1038/nature09676 21307940 PMC4025995

[B59] Philippe H. Poustka A. J. Chiodin M. Hoff K. J. Dessimoz C. Tomiczek B. (2019). Mitigating anticipated effects of systematic errors supports sister-group relationship between Xenacoelomorpha and Ambulacraria. *Curr. Biol.* 29 1818–1826.e6. 10.1016/j.cub.2019.04.009 31104936

[B60] Roller L. Čižmár D. Bednár B. Žitňan D. (2016). Expression of RYamide in the nervous and endocrine system of *Bombyx mori*. *Peptides* 80 72–79. 10.1016/j.peptides.2016.02.003 26896568

[B61] Rouse G. W. Wilson N. G. Carvajal J. I. Vrijenhoek R. C. (2016). New deep-sea species of *Xenoturbella* and the position of Xenacoelomorpha. *Nature* 530 94–97. 10.1038/nature16545 26842060

[B62] Rowe M. L. Achhala S. Elphick M. R. (2014). Neuropeptides and polypeptide hormones in echinoderms: new insights from analysis of the transcriptome of the sea cucumber *Apostichopus japonicus*. *Gen. Comp. Endocrinol.* 197 43–55. 10.1016/j.ygcen.2013.12.002 24345384

[B63] Schaefer M. Picciotto M. R. Kreiner T. Kaldany R. R. Taussig R. Scheller R. H. (1985). *Aplysia* neurons express a gene encoding multiple FMRFamide neuropeptides. *Cell* 41 457–467. 10.1016/s0092-8674(85)80019-2 3838698

[B64] Semmens D. C. Mirabeau O. Moghul I. Pancholi M. R. Wurm Y. Elphick M. R. (2016). Transcriptomic identification of starfish neuropeptide precursors yields new insights into neuropeptide evolution. *Open Biol.* 6:150224. 10.1098/rsob.150224 26865025 PMC4772807

[B65] Shyamala M. Fisher J. M. Scheller R. H. (1986). A neuropeptide precursor expressed in *Aplysia* neuron L5. *DNA* 5 203–208. 10.1089/dna.1986.5.203 3013547

[B66] Smith M. K. Wang T. Suwansa-Ard S. Motti C. A. Elizur A. Zhao M. (2017). The neuropeptidome of the crown-of-thorns starfish, *Acanthaster planci*. *J. Proteomics* 165 61–68. 10.1016/j.jprot.2017.05.026 28577918

[B67] Stemmler E. A. Bruns E. A. Gardner N. P. Dickinson P. S. Christie A. E. (2007). Mass spectrometric identification of pEGFYSQRYamide: a crustacean peptide hormone possessing a vertebrate neuropeptide Y (NPY)-like carboxy-terminus. *Gen. Comp. Endocrinol.* 152 1–7. 10.1016/j.ygcen.2007.02.025 17420018 PMC1950731

[B68] Suwansa-Ard S. Chaiyamoon A. Talarovicova A. Tinikul R. Tinikul Y. Poomtong T. (2018). Transcriptomic discovery and comparative analysis of neuropeptide precursors in sea cucumbers (Holothuroidea). *Peptides* 99 231–240. 10.1016/j.peptides.2017.10.008 29054501

[B69] Telford M. J. Copley R. R. (2016). Zoology: war of the worms. *Curr. Biol.* 26 R335–R337. 10.1016/j.cub.2016.03.015 27115693

[B70] Tensen C. P. Cox K. J. Smit A. B. van der Schors R. C. Meyerhof W. Richter D. (1998). The lymnaea cardioexcitatory peptide (LyCEP) receptor: a G-protein-coupled receptor for a novel member of the RFamide neuropeptide family. *J. Neurosci.* 18 9812–9821. 10.1523/jneurosci.18-23-09812.1998 9822740 PMC6793288

[B71] Thiel D. Franz-Wachtel M. Aguilera F. Hejnol A. (2018). Xenacoelomorph neuropeptidomes reveal a major expansion of neuropeptide systems during early bilaterian evolution. *Mol. Biol. Evol.* 35 2528–2543. 10.1093/molbev/msy160

[B72] Tian S. Zandawala M. Beets I. Baytemur E. Slade S. E. Scrivens J. H. (2016). Urbilaterian origin of paralogous GnRH and corazonin neuropeptide signalling pathways. *Sci. Rep.* 6:28788. 10.1038/srep28788 27350121 PMC4923880

[B73] Tinoco A. B. Semmens D. C. Patching E. C. Gunner E. F. Egertová M. Elphick M. R. (2018). Characterization of NGFFYamide signaling in starfish reveals roles in regulation of feeding behavior and locomotory systems. *Front. Endocrinol.* 9:507. 10.3389/fendo.2018.00507 30283399 PMC6156427

[B74] Trifinopoulos J. Nguyen L.-T. von Haeseler A. Minh B. Q. (2016). W-IQ-TREE: a fast online phylogenetic tool for maximum likelihood analysis. *Nucleic Acids Res.* 44 W232–W235. 10.1093/nar/gkw256 27084950 PMC4987875

[B75] Ubuka T. Tsutsui K. (2018). Comparative and evolutionary aspects of gonadotropin-inhibitory hormone and FMRFamide-like peptide systems. *Front. Neurosci.* 12:747. 10.3389/fnins.2018.00747 30405335 PMC6200920

[B76] Veenstra J. A. (2011). Neuropeptide evolution: neurohormones and neuropeptides predicted from the genomes of *Capitella teleta* and *Helobdella robusta*. *Gen. Comp. Endocrinol.* 171 160–175. 10.1016/j.ygcen.2011.01.005 21241702

[B77] Veenstra J. A. (2015). The power of next-generation sequencing as illustrated by the neuropeptidome of the crayfish *Procambarus clarkii*. *Gen. Comp. Endocrinol.* 224 84–95. 10.1016/j.ygcen.2015.06.013 26149328

[B78] Veenstra J. A. Khammassi H. (2017). Rudimentary expression of RYamide in *Drosophila melanogaster* relative to other *Drosophila* species points to a functional decline of this neuropeptide gene. *Insect Biochem. Mol. Biol.* 83 68–79. 10.1016/j.ibmb.2017.03.001 28286046

[B79] Wood N. J. Mattiello T. Rowe M. L. Ward L. Perillo M. Arnone M. I. (2018). Neuropeptidergic systems in pluteus larvae of the sea urchin *Strongylocentrotus purpuratus*: neurochemical complexity in a “simple” nervous system. *Front. Endocrinol.* 9:628. 10.3389/fendo.2018.00628 30410468 PMC6209648

[B80] Yañez-Guerra L. A. Delroisse J. Barreiro-Iglesias A. Slade S. E. Scrivens J. H. Elphick M. R. (2018). Discovery and functional characterisation of a luqin-type neuropeptide signalling system in a deuterostome. *Sci. Rep.* 8:7220. 10.1038/s41598-018-25606-2 29740074 PMC5940834

[B81] Zandawala M. Moghul I. Yañez Guerra L. A. Delroisse J. Abylkassimova N. Hugall A. F. (2017). Discovery of novel representatives of bilaterian neuropeptide families and reconstruction of neuropeptide precursor evolution in ophiuroid echinoderms. *Open Biol.* 7:170129. 10.1098/rsob.170129 28878039 PMC5627052

[B82] Zandawala M. Tian S. Elphick M. R. (2018). The evolution and nomenclature of GnRH-type and corazonin-type neuropeptide signaling systems. *Gen. Comp. Endocrinol.* 264 64–77. 10.1016/j.ygcen.2017.06.007 28622978

