# Supplementary material for: Evolution and Comparative Physiology of Luqin-Type Neuropeptide Signaling
Source: Front Neurosci. 2020 Feb 18;14:130. doi: 10.3389/fnins.2020.00130 (PMC7041311; doi:10.3389/fnins.2020.00130)
Supplement: Supplementary file 1 [file Image_1.PDF]

A

Daphnia pulex RYamide precursor

MARKESVFWLFCTLALMMSVVLVDAQTFFTNGRYGKRSEVRSRVASRSADERFFGGPRFGRSGNGGIVLGNSELDARNPERFFI
GSRYGKRSEMEQIVPSPQVDESTSNSQEKETFLECNPIGIEQLYHCIERLKSAHHFDLMQHQQV

Proasellus cavaticus RYamide precursor

MFFLRSFLLLVALGTLEMTLGQGFYSTRYGKRTNDASSSSNTGYENEGHSGFYANRYGRSSDLPEIKIRSSRFIGGSRYGKRST
TTPEGDLPRNIDGDTFDCVMVNSPSLYRCLRKSFQPSEETIN

Zootermopsis nevadensis RYamide precursor

MASASSVVILIMLVTCSLVTLALSAQFYTSGRYGKRDLAQRSMFWSGSRYGRSSGGGGGRRQGGNNPVEVAVRNDRFFIGSRYG
KRSEEPLTTTDETVGVLVPTEDTNSQVACMYTGVANLYRCYKRKGNSSEDASSEHE

Nasonia vitripennis RYamide precursor

MISSSRKIRRVSDYLKLDKLIVWLWISGIFLTLVSSQDNFYASGRFGKRKYALSMSQIPLCSKFDRSEDRSAGNSLKDSSLFSS
ARFGRSEDRNTGNSLRDSSSFFPARYGRSEDRSTGNSLRDSSSFFPARFGRSEDRSTGNSLKDSSSFSPARYGRSEDRSSGNSL
KESSFFSPGRYGRSEGHKNPKELPKFFEIKPRVDQFFIGSRYGKRSLSMLEPQPPLEALHNQRFEAAIDYLDRIKQNLAEAEEI
EDETRDASRDDELVEAIYPNDYTGLSKI

B

Echinococcus multilocularis Luqin-type precursor

MRGTFISILTLFYFASSLRLHDYDGEAEPTVSAATPVGEAEDDIDFFPPRYGVAKRYPLLSDLDEGMMIENPYWAKEISRRSPQ
FAWRPHSRFGRR

Echinococcus granulosus Luqin-type precursor

MRGTFISLLTLFYFASSLRLHDYDGEAEPAVSAATPVGEAEDDIDSLPPRYRVAKRYPLLSDLDEEMMIENPYWAKEISRRSPQ
FAWRPHSRFGRR

Taenia solium Luqin-type precursor

MRATCISLLTLFYFASSLRLHDLEGPVEPTVSSAMPVGEPEDDIDFPLPSRYRLAKRYPHLSNFDEEMVIGNPYWGKEISRRSP
QFAWRPHSRFGRR

Mesocestoides corti Luqin-type precursor

MVSCLTETSVILMVLLCFASALRLREPEADLDDEDIDFVVPPPLRFHKRFPMMGEFGDGAMWSPMGEREVARRSAHFAWRPHSR
FGRR

Supplementary Figure 1. Luqin-type precursor proteins with atypical characteristics. Luqin-type precursors typically comprise one or two luqin-type neuropeptides, which are located immediately after the N-terminal signal peptide, and a pair of cysteine residues separated by ten amino acid residues located in the C-terminal region of the precursor (see Figure 1 of this paper). The sequences of luqin-type precursors with atypical characteristics are shown in this figure, with the signal peptide shown in blue, potential mono/dibasic cleavage sites shown in green and predicted neuropeptides shown in red. Peptide products of the precursors that have been identified using mass spectrometry are highlighted in yellow. Where a C-terminal pair of cysteine residues separated by ten amino-acid residues are present, this region of the precursor is underlined.

A. Arthropod RYamide precursors that contain more than two luqin-type neuropeptides. The RYamide precursor in the crustacean *D. pulex* comprises three predicted neuropeptides, two of which have been identified by mass spectrometry. The first peptide is located immediately after the signal peptide and is subject to post-translational conversion of the C-terminal glycine to an amide group (as in other luqin-type peptides) and conversion of the N-terminal glutamine residue to pyroglutamate. Two different forms of the third neuropeptide are detected by mass spectrometry - a twenty-seven residue peptide and a C-terminal fragment of this peptide generated by cleavage at the highlighted arginine residue in the longer peptide. The precursor has a canonical C-terminal pair of cysteine residues separated by ten amino-acid residues. The RYamide precursors in the crustacean *Proasellus cavaticus* (isopod) and in the insect *Zootermopsis nevadensis* (termite) also comprise three putative luqin-type neuropeptides but the structures of these peptides have not been confirmed using mass spectrometry. The RYamide precursor in the insect *Nasonia vitripennis* (parasitoid wasp) is remarkable in comprising seven putative luqin-type neuropeptides, but only the first of these has been identified using mass spectrometry. The six other putative luqin-type neuropeptides are bounded by predicted mono/dibasic cleavage sites and also contain potential internal monobasic cleavage sites. Note also that the precursor lacks a C-terminal region containing the two cysteines separated by ten amino acid residues.

B. **Luqin-type precursors with a C-terminally located neuropeptide in parasitic platyhelminths.** The sequences of the luqin-type precursors from four cestode species are shown and they all have the atypical characteristics of comprising a single luqin-type neuropeptide located at the C-terminus and lacking a C-terminal pair of cysteines separated by ten amino acid residues. In spite of these differences at the precursor level, the neuropeptides derived from these precursors exhibit a high level of sequence conservation with luqin-type neuropeptides from other spiralians. Thus, the WRPHSRF-NH_2 motif in these peptides is structurally similar to the WRQPGRF-NH_2 motif of luqin-type neuropeptides in molluscs, annelids, brachiopods and nemerteans. The sequence data and mass spectroscopic data shown in this figure have been reported in the following publications: (27,33,34,38,45).
